# What to Do When the Oceans Rise

**DOI:** 10.1371/journal.pbio.1001387

**Published:** 2012-09-04

**Authors:** Lauren-Kristine Pryzant, John F. Bruno

**Affiliations:** 1 Kenan-Flagler Business School, The University of North Carolina at Chapel Hill, Chapel Hill, North Carolina, United States of America; 2 Department of Biology, The University of North Carolina at Chapel Hill, Chapel Hill, North Carolina, United States of America

## Abstract

Lauren-Kristine Pryzant and John Bruno review *Adapting to a Changing Environment: Confronting the Consequences of Climate Change.*

**Figure:**
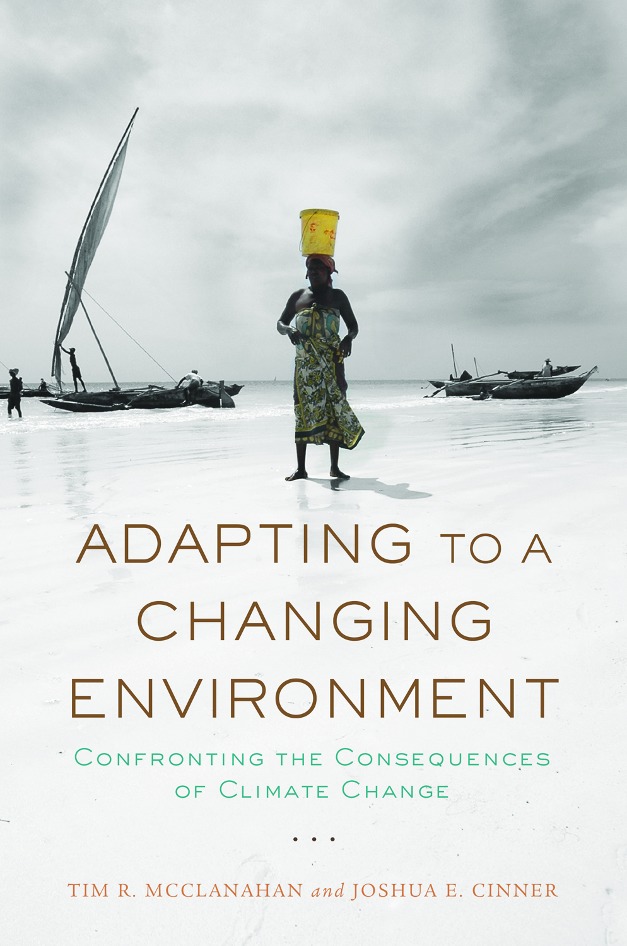
McClanahan TR, Cinner J (2011) Adapting to a Changing Environment: Confronting the Consequences of Climate Change. New York: Oxford University Press, Inc. 208 p. ISBN 978-0199754489 (hardcover). US$65.00.

**Figure:**
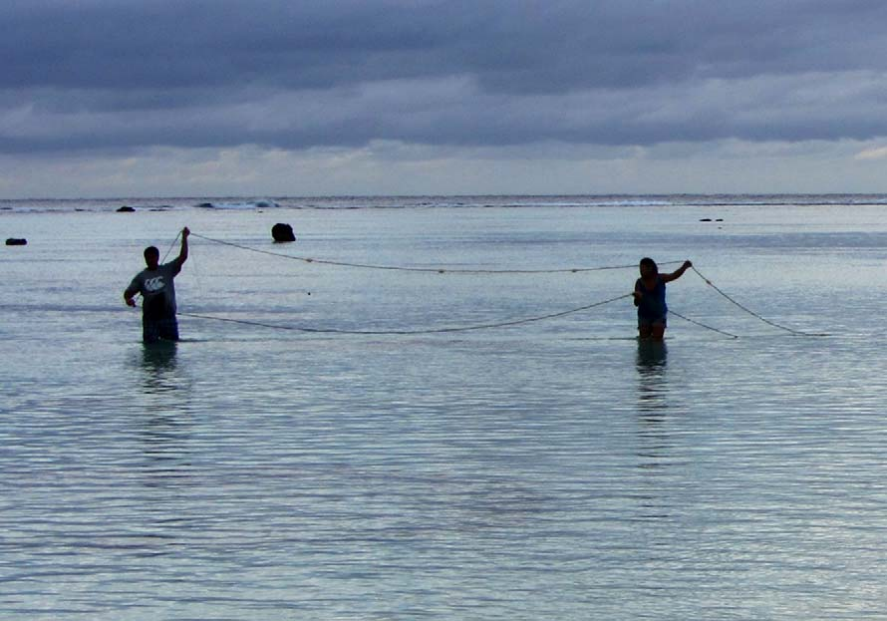
Net fishing on Aitutaki Island, Cook Islands. Coral reef fisheries like this are threatened by climate change. Photo: Lauren-Kristine Pryzant.

This summer, Americans are experiencing climate change as record-breaking heat and drought. Our new normal, with its massive wildfires and severe storms, has given the whole nation a sense of the economic and social consequences of global warming that coastal communities around the world have been experiencing for decades.

If you live near the sea, you're probably witnessing the consequences of too much carbon dioxide in the atmosphere as rising sea level, coastal flooding, and eroding shorelines. In the western Pacific and Indian Oceans, low-lying islands are being engulfed. Meanwhile, in some parts of the Arctic, the rate of erosion has doubled to tens of meters per year due to thawing and the loss of sea, which increases wind fetch. Rising seas also ruin coastal farmland and fresh water aquifers and can destroy biologically rich habitats like marshlands and mangroves.

The costs of either rebuilding or relocating in response are enormous but unavoidable. Furthermore, since the economies of many coastal communities are based on fisheries and tourism, the impacts of anthropogenic climate change threaten their long-term sustainability [1]. Given their vulnerability, coastal communities are on the front line of global warming. But do they have the capacity to adapt to so much environmental change? Do their responses to past challenges suggest strategies for coping with future change? Can we predict which communities are most vulnerable and help them to become more resilient?

To answer these questions, scientists like Tim McClanahan and Joshua Cinner are merging marine and climate change ecology with modern social science. Their goal is to figure out what aspects of coastal communities facilitate social adaptation and how these traits can be promoted. McClanahan is a renowned coral reef ecologist who lives in Kenya and works for the Wildlife Conservation Society. Cinner, a Research Fellow based at James Cook University in Townsville, Australia, is at the forefront of a revolution in the social sciences. He's leading the way to replace the traditional descriptive case study approach with a sort of human macroecology [2], where relationships between societies and their environment are explored by assembling geographic databases of social and ecological traits. This new approach is inherently large scale and statistical as opposed to the traditional local-qualitative method.

McClanahan and Cinner's new book, *Adapting to a Changing Environment: Confronting the Consequences of Climate Change*, is a primer and also an application of this emerging holistic science. The book is concise, accessible, and written for students, scientists from other disciplines, and policy makers. The authors use coastal east Africa as a case study to develop their model of estimating the “social adaptive capacity” of communities. Unlike temperate and polar coasts, the shorelines of much of the tropics are fringed by coral reefs that buffer coastal communities from waves and storms, provide productive fisheries, and are the base of a tourism economy. The downside is that corals are quite sensitive to ocean warming, and their ongoing global disappearance [3] is both depriving people of their livelihoods and simultaneously increasing erosion as their buffering function is lost [4]. In other words, the vulnerability of this threatened ecosystem is passed on to its human dependents. (Such interconnectedness is a recurrent theme in the book.)

The book contains thorough but understandable introductory chapters on marine fisheries, climate change, and coral reefs. McClanahan and Cinner highlight the challenges of effective fishery management and describe actions taken on international and national levels to more effectively and sustainably manage marine resources. A chapter on coral reef resilience explains what a coral is (a coelentrate, related to jellyfish), how corals form reefs, and the role of the microscopic zooxanthellae that form a crucial symbiosis with their coral hosts. The authors explain how this symbiosis can be disrupted by small temperature increases, leading to the eviction of the zooxanthellae and the “bleaching” and death of the coral if temperatures remain elevated too long. Coral mortality in turn disrupts fisheries as fish habitat is lost and can wipe out tourism based on SCUBA diving. The chapter also includes a refreshingly honest assessment about what local managers can do to make reefs more resilient to climate change (very little [5]).

Coastal communities have a long history of dealing with disturbances such as cyclones. Social adaptive capacity—the ability of a community to respond effectively to change—can be influenced by social traits such as occupational and institutional flexibility, literacy, household assets, infrastructure, and even the degree of social organization. And it's this capacity, and how to measure it, that lies at the heart of the book. Enhancing the adaptive capacity is the ultimate goal and where global nongovernmental organizations like the World Bank are focusing. However, such social engineering is always easier said than done; even with the best of intentions, it often simply creates more poverty, hardship, and dependency [6].

The next step will be testing what is currently a predictive framework; measuring the rate and then the success of adaptive actions by communities and nations and then asking, retrospectively, which characteristics were the best predictors of social adaptive capacity and which responses were most effective. Unfortunately, actually testing the efficacy of policies designed to limit the impacts of climate change on ecosystems and people is rarely done. And even when an approach is found to be ineffective [5], advocates rarely stop promoting it, such as the idea that the implementation of marine protected areas makes reefs more resilient to ocean warming and acidification. Even more challenging is the prospect of identifying which indicators are actually causally linked with adaptive capacity and which merely co-vary with the traits that actually confer resilience.

Having the capacity to adapt is one thing—actually using it is quite another. Here in North Carolina, where our adaptive capacity index would be off the charts in relation to anything in the Indian Ocean, politicians that don't believe in climate change recently introduced legislation that would effectively block coastal counties' attempts at adaptive planning in response to sea level rise. They were lobbied by real estate investment groups who fear that any form of state-sanctioned adaptation would hamper coastal development and depress real estate values. Ironically, the northern shore of North Carolina was just found to be within a global hot spot of sea level rise [7], where sea level is expected to increase by as much as a meter or more this century, whether or not we plan for it.

Of course, the ultimate strategy for adapting to global warming is to rapidly and radically reduce greenhouse gas emissions, i.e., mitigation. The difficulty of this is obvious and has led many scientists and activists to move on to adaptation, which from a distance seems less complex and more likely to actually happen. But the same forces that have blocked mitigation (e.g., denial, short-term economic thinking, etc.) could just as easily trip up adaptive planning.

The African nations where McClanahan and Cinner work emit relatively little carbon dioxide per capita; therefore, mitigation at a global scale is largely beyond reach. For these nations, adaption is the only means to cope with a changing climate. Whether they can build their adaptive capacity quickly enough to meet the challenges ahead remains to be seen. But we'd all do well to start building the adaptive capacity of our own communities, because the front lines of climate change appear to be accelerating.

**About the Authors**

Lauren-Kristine Pryzant is an undergraduate and Morehead-Cain scholar studying business administration and economics in the Kenan-Flagler Business School at UNC. John Bruno is a marine ecologist and professor at UNC. His research is focused on marine biodiversity and the impacts of climate change on marine ecosystems. John earned his PhD from Brown University in ecology and evolutionary biology and is currently working across the Caribbean on coral reef ecology and conservation.
